# Dioxidobis(pentane-2,4-dionato-κ^2^ *O*,*O*′)(pyridine-4-carbaldehyde oxime-κ*N* ^1^)uranium(VI)

**DOI:** 10.1107/S1600536808012889

**Published:** 2008-05-10

**Authors:** Takeshi Kawasaki, Takafumi Kitazawa

**Affiliations:** a Department of Chemistry, Faculty of Science, Toho University, 2-2-1 Miyama, Funabashi, Chiba 274-8510, Japan; b Research Center for Materials with Integrated Properties, Toho University, Miyama, Funabashi, Chiba 274-8510, Japan

## Abstract

The title compound, [U(C_5_H_7_O_2_)_2_O_2_(C_6_H_6_N_2_O)], exhibits a penta­gonal–bipyramidal coordination geometry around the U^VI^ atom, involving two bidentate acetyl­acetonate ions and the pyridine ring of the pyridine-4-carbaldehyde oxime ligand. Hydrogen bonds exist between the OH group of the pyridine-4-carbaldehyde oxime ligand and the two O atoms of the acetyl­acetonate ions.

## Related literature

For related literature, see: Alcock *et al.* (1984 ▶, 1987 ▶); Kawasaki *et al.* (2006 ▶); Saeki *et al.* (2006 ▶).
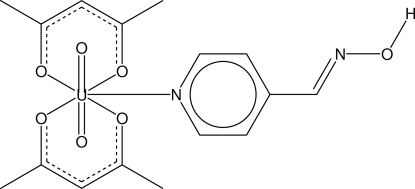


## Experimental

### 

#### Crystal data

[U(C_5_H_7_O_2_)_2_O_2_(C_6_H_6_N_2_O)]
*M* *_r_* = 590.37
Triclinic, 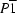

*a* = 8.1969 (6) Å
*b* = 11.2632 (9) Å
*c* = 11.7448 (9) Å
α = 71.016 (1)°
β = 75.660 (2)°
γ = 80.137 (2)°
*V* = 988.51 (13) Å^3^
*Z* = 2
Mo *K*α radiation
μ = 8.25 mm^−1^
*T* = 291 K
0.20 × 0.18 × 0.15 mm

#### Data collection

Bruker SMART CCD area-detector diffractometer
Absorption correction: multi-scan (*SADABS*; Sheldrick, 1996 ▶) *T*_min_ = 0.289, *T*_max_ = 0.371 (expected range = 0.226–0.290)
7404 measured reflections
4832 independent reflections
4538 reflections with *I* > 2σ(*I*)
*R*_int_ = 0.013

#### Refinement

*R*[*F* ^2^ > 2σ(*F* ^2^)] = 0.019
*wR*(*F* ^2^) = 0.048
*S* = 1.09
4832 reflections
240 parameters
H-atom parameters constrained
Δρ_max_ = 0.63 e Å^−3^
Δρ_min_ = −0.71 e Å^−3^

### 

Data collection: *SMART* (Bruker, 2001 ▶); cell refinement: *SAINT* (Bruker, 2001 ▶); data reduction: *SAINT*; program(s) used to solve structure: *SHELXS97* (Sheldrick, 2008 ▶); program(s) used to refine structure: *SHELXL97* (Sheldrick, 2008 ▶); molecular graphics: *SHELXTL* (Sheldrick, 2008 ▶) and *CrystalMaker* (*CrystalMaker*, 2007 ▶); software used to prepare material for publication: *SHELXTL*.

## Supplementary Material

Crystal structure: contains datablocks I, global. DOI: 10.1107/S1600536808012889/im2063sup1.cif

Structure factors: contains datablocks I. DOI: 10.1107/S1600536808012889/im2063Isup2.hkl

Additional supplementary materials:  crystallographic information; 3D view; checkCIF report

## Figures and Tables

**Table 1:** Hydrogen-bond geometry (Å, °)

*D*—H⋯*A*	*D*—H	H⋯*A*	*D*⋯*A*	*D*—H⋯*A*
O7—H7⋯O5^i^	0.82	2.49	3.018 (4)	123
O7—H7⋯O3^i^	0.82	2.29	3.083 (4)	163

Symmetry code: (i) 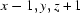.

**Fig. 1.**
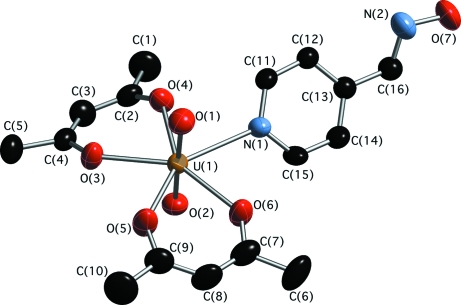
Molecular structure of [UO2(acac)2(4-aldpy)] (I) showing the atomic notations; displacement ellipsoids are depicted at the 50% probability level; H atoms are omitted for clarity.

**Fig. 2.**
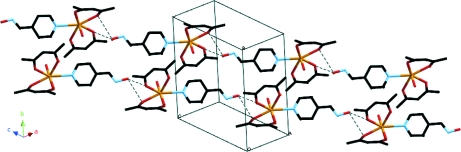
Strcuture of the 1-D chain aggregate of I. Dashed lines indicate intermolecular OH···Oacac hydrogen bonds between neighboring molecules; H atoms are omitted for clarity.

## supplementary crystallographic information

### Comment

Actinoide chemistry is highly related to the reprocessing of nuclear fuels and treatment of actinoide wastes in the backend chemistry of today's operating nuclear power plants. The fundamental investigation of the bonding and structure of uranium complexes provides important information in the field of backend chemistry. Structural properties of [AnO_2_(acac)_2_(py)] complexes (An = U, Np) (Alcock *et al.*, 1984; Alcock *et al.*, 1987; Kawasaki *et al.*, 2006) were reported. [AnO_2_(acac)_2_(py)] complexes exhibit pentagonal-bipyramidal geometry around the An^VI^ ion which are coordinated by two oxo ligands, four oxygen atoms from the acac ions and one nitrogen atom from the pyridine molecule. Recently, ^237^Np Mößbauer spectra of the [NpO_2_(acac)_2_(py)] (Kawasaki *et al.*, 2006; Saeki *et al.*, 2006) were reported. We report herein the synthesis and crystal structure of the new uranyl(VI) acetylacetonate complex [UO_2_(acac)_2_(4-aldpy)], (I), (4-aldpy = pyridine-4-carbaldehyde oxime).

In the title complex, [UO_2_(acac)_2_(4-aldpy)] (I), the uranyl(VI) moiety is constructed from U1, O1 and O2. The O1—U1—O2 angle of the uranyl(VI) ion is 177.7 (1) °. U1 exhibits a pentagonal-bipyramidal coordination geometry. The two O atoms from the uranyl(VI) ion occupy the U1 axial positions whereas four O atoms from the two chelating acac ions and one N atom from the 4-aldpy are situated in the equatorial plane (Fig. 1). The deviations of the four O atoms (O3, O4, O5 and O6) of the acac and one N1 atom of the 4-aldpy from the equatorial plane (O3, O4, O5, O6 and N1) are within 0.13 Å. The dihedral angle between the pyridine ring of the 4-aldpy ligand and the equatorial plane of the uranyl(VI) ion in I is 44.5 (1)°. The U1—O_acac_ distances are longer than the U1—O_uranyl_ distances and are shorter than the U1—N1 distance which measures to 2.599 (3) Å. This bond length is similar to the U—N distance [2.602 (3) Å] in [UO_2_(acac)_2_(py)] (Kawasaki *et al.*). However, [UO_2_(acac)_2_(py)] crystallized in the non-centrosymmetric space group, *Fdd2*, whereas I crystallized in the centrosymmetric space group *P*1. The differences in the crystal structures are obviously caused by the additional aldoxime substituent in I acting as an efficient hydrogen bond donor site. The O7 atom of the OH group of the 4-aldpy is connected with O3 and O5 atoms of the acac by intermolecular hydrogen bonds. This results in a 1-D chain aggregate of I along the [1, 0, - 1] direction (Fig. 2).

### Experimental

To 10 ml of a methanolic solution containing 1 mmol UO_2_(NO_3_)_2_.6H_2_O was added 3.0 mmol of acetylacetone and 3.0 mmol of pyridine-4-carbaldehyde oxime oximepyridine in 5 ml of methanol. After the solvent evaporated slowly at room temperature for a few days, orange crystals of the title complex were obtained.

### Refinement

All H atoms were placed at calculated positions (O—H = 0.82 Å, C(CH)—H = 0.93 Å or C(CH_3_)—H = 0.96 Å) and allowed to ride on the parent atom [*U*_iso_(H) = 1.2*U*_eq_(CH) or *U*_iso_(H) = 1.5*U*_eq_(CH_3_, O)].

### Crystal data

**Table:**

[U(C_5_H_7_O_2_)_2_O_2_(C_6_H_6_N_2_O)]	*Z* = 2
*M**_r_* = 590.37	*F*_000_ = 556
Triclinic, *P*1	*D*_x_ = 1.983 Mg m^−^^3^
Hall symbol: -P 1	Mo *K*α radiation λ = 0.71073 Å
*a* = 8.1969 (6) Å	Cell parameters from 5400 reflections
*b* = 11.2632 (9) Å	θ = 2.3–28.3º
*c* = 11.7448 (9) Å	µ = 8.25 mm^−^^1^
α = 71.016 (1)º	*T* = 291 K
β = 75.660 (2)º	Block, orange
γ = 80.137 (2)º	0.20 × 0.18 × 0.15 mm
*V* = 988.51 (13) Å^3^	

### Data collection

**Table:**

Bruker SMART CCD area-detector diffractometer	4832 independent reflections
Radiation source: fine-focus sealed tube	4538 reflections with *I* > 2σ(*I*)
Monochromator: graphite	*R*_int_ = 0.013
Detector resolution: 8.366 pixels mm^-1^	θ_max_ = 28.3º
*T* = 291 K	θ_min_ = 1.9º
φ and ω scans	*h* = −10→9
Absorption correction: multi-scan(SADABS; Sheldrick, 1996)	*k* = −14→15
*T*_min_ = 0.289, *T*_max_ = 0.371	*l* = −15→15
7404 measured reflections	

### Refinement

**Table:**

Refinement on *F*^2^	Secondary atom site location: difference Fourier map
Least-squares matrix: full	Hydrogen site location: inferred from neighbouring sites
*R*[*F*^2^ > 2σ(*F*^2^)] = 0.019	H-atom parameters constrained
*wR*(*F*^2^) = 0.048	*w* = 1/[σ^2^(*F*_o_^2^) + (0.018*P*)^2^ + 0.4544*P*] where *P* = (*F*_o_^2^ + 2*F*_c_^2^)/3
*S* = 1.09	(Δ/σ)_max_ = 0.016
4832 reflections	Δρ_max_ = 0.63 e Å^−^^3^
240 parameters	Δρ_min_ = −0.71 e Å^−^^3^
Primary atom site location: structure-invariant direct methods	Extinction correction: none

### Special details

**Table:**

Geometry. All e.s.d.'s (except the e.s.d. in the dihedral angle between two l.s. planes) are estimated using the full covariance matrix. The cell e.s.d.'s are taken into account individually in the estimation of e.s.d.'s in distances, angles and torsion angles; correlations between e.s.d.'s in cell parameters are only used when they are defined by crystal symmetry. An approximate (isotropic) treatment of cell e.s.d.'s is used for estimating e.s.d.'s involving l.s. planes.
Refinement. Refinement of *F*^2^ against ALL reflections. The weighted *R*-factor *wR* and goodness of fit *S* are based on *F*^2^, conventional *R*-factors *R* are based on *F*, with *F* set to zero for negative *F*^2^. The threshold expression of *F*^2^ > σ(*F*^2^) is used only for calculating *R*-factors(gt) *etc*. and is not relevant to the choice of reflections for refinement. *R*-factors based on *F*^2^ are statistically about twice as large as those based on *F*, and *R*- factors based on ALL data will be even larger.

### Fractional atomic coordinates and isotropic or equivalent isotropic displacement parameters (Å^2^)

**Table:**

	*x*	*y*	*z*	*U*_iso_*/*U*_eq_	
U1	0.097943 (12)	0.796870 (9)	0.824753 (9)	0.03536 (4)	
O1	−0.0310 (3)	0.9423 (2)	0.7952 (2)	0.0525 (6)	
O2	0.2248 (3)	0.6515 (2)	0.8605 (2)	0.0503 (5)	
O3	0.3515 (3)	0.8856 (2)	0.7061 (2)	0.0499 (5)	
O4	0.2204 (3)	0.8617 (2)	0.9494 (2)	0.0490 (5)	
O5	0.1115 (3)	0.7877 (3)	0.6262 (2)	0.0601 (7)	
O6	−0.1153 (3)	0.6784 (3)	0.8304 (2)	0.0579 (6)	
O7	−0.5265 (4)	0.6725 (3)	1.5844 (2)	0.0731 (8)	
H7	−0.5787	0.7280	1.6141	0.110*	
N1	−0.0878 (3)	0.7423 (2)	1.0457 (2)	0.0420 (5)	
N2	−0.4406 (4)	0.7272 (3)	1.4648 (3)	0.0593 (8)	
C1	0.4046 (6)	0.8379 (5)	1.0811 (4)	0.0735 (12)	
H1A	0.3173	0.8863	1.1233	0.110*	
H1B	0.5126	0.8648	1.0740	0.110*	
H1C	0.4033	0.7501	1.1266	0.110*	
C2	0.3743 (4)	0.8576 (3)	0.9552 (3)	0.0470 (7)	
C3	0.5049 (4)	0.8757 (4)	0.8536 (3)	0.0531 (8)	
H3	0.6133	0.8728	0.8664	0.064*	
C4	0.4875 (4)	0.8978 (3)	0.7336 (3)	0.0442 (7)	
C5	0.6322 (5)	0.9391 (4)	0.6284 (4)	0.0598 (9)	
H5A	0.6415	0.8938	0.5701	0.090*	
H5B	0.7351	0.9220	0.6584	0.090*	
H5C	0.6127	1.0279	0.5890	0.090*	
C6	−0.2910 (7)	0.5394 (5)	0.8211 (5)	0.0811 (14)	
H6A	−0.2656	0.4802	0.8959	0.122*	
H6B	−0.2988	0.4946	0.7660	0.122*	
H6C	−0.3968	0.5882	0.8394	0.122*	
C7	−0.1524 (5)	0.6261 (3)	0.7615 (4)	0.0546 (9)	
C8	−0.0769 (6)	0.6479 (4)	0.6384 (4)	0.0612 (10)	
H8	−0.1122	0.6050	0.5946	0.073*	
C9	0.0470 (5)	0.7286 (4)	0.5752 (3)	0.0551 (8)	
C10	0.1107 (7)	0.7529 (6)	0.4392 (4)	0.0844 (14)	
H10A	0.0741	0.8381	0.3971	0.127*	
H10B	0.0667	0.6955	0.4119	0.127*	
H10C	0.2321	0.7406	0.4216	0.127*	
C11	−0.1242 (4)	0.8257 (3)	1.1105 (3)	0.0454 (7)	
H11	−0.0868	0.9054	1.0725	0.054*	
C12	−0.2131 (4)	0.7997 (3)	1.2294 (3)	0.0450 (7)	
H12	−0.2366	0.8607	1.2704	0.054*	
C13	−0.2680 (4)	0.6800 (3)	1.2880 (3)	0.0422 (6)	
C14	−0.2309 (5)	0.5944 (3)	1.2224 (3)	0.0499 (8)	
H14	−0.2650	0.5136	1.2590	0.060*	
C15	−0.1426 (4)	0.6286 (3)	1.1019 (3)	0.0477 (7)	
H15	−0.1205	0.5699	1.0582	0.057*	
C16	−0.3646 (4)	0.6443 (3)	1.4146 (3)	0.0499 (7)	
H16	−0.3700	0.5594	1.4585	0.060*	

### Atomic displacement parameters (Å^2^)

**Table:**

	*U*^11^	*U*^22^	*U*^33^	*U*^12^	*U*^13^	*U*^23^
U1	0.03482 (6)	0.03467 (6)	0.03998 (6)	−0.00562 (4)	−0.00817 (4)	−0.01417 (4)
O1	0.0479 (13)	0.0446 (13)	0.0623 (14)	0.0015 (10)	−0.0158 (11)	−0.0122 (11)
O2	0.0508 (13)	0.0390 (11)	0.0602 (14)	0.0021 (10)	−0.0093 (11)	−0.0185 (10)
O3	0.0451 (12)	0.0615 (14)	0.0453 (12)	−0.0194 (11)	−0.0089 (10)	−0.0120 (10)
O4	0.0425 (12)	0.0625 (14)	0.0517 (13)	−0.0112 (10)	−0.0082 (10)	−0.0279 (11)
O5	0.0639 (15)	0.0816 (19)	0.0458 (13)	−0.0301 (14)	−0.0099 (11)	−0.0229 (12)
O6	0.0551 (14)	0.0736 (17)	0.0558 (14)	−0.0292 (13)	−0.0066 (11)	−0.0255 (12)
O7	0.081 (2)	0.081 (2)	0.0483 (14)	−0.0147 (16)	0.0146 (13)	−0.0240 (14)
N1	0.0463 (14)	0.0372 (12)	0.0438 (13)	−0.0086 (11)	−0.0025 (11)	−0.0166 (10)
N2	0.0593 (18)	0.068 (2)	0.0463 (15)	−0.0052 (15)	0.0015 (13)	−0.0211 (14)
C1	0.076 (3)	0.098 (3)	0.061 (2)	−0.014 (2)	−0.027 (2)	−0.030 (2)
C2	0.0491 (18)	0.0439 (16)	0.0576 (19)	−0.0041 (14)	−0.0196 (15)	−0.0219 (14)
C3	0.0380 (16)	0.065 (2)	0.063 (2)	−0.0031 (15)	−0.0154 (15)	−0.0240 (17)
C4	0.0367 (15)	0.0370 (15)	0.0599 (19)	−0.0051 (12)	−0.0083 (13)	−0.0162 (13)
C5	0.0427 (18)	0.066 (2)	0.068 (2)	−0.0156 (16)	−0.0021 (16)	−0.0179 (19)
C6	0.088 (3)	0.074 (3)	0.092 (3)	−0.043 (3)	−0.037 (3)	−0.009 (2)
C7	0.056 (2)	0.0462 (18)	0.070 (2)	−0.0095 (15)	−0.0324 (18)	−0.0129 (16)
C8	0.081 (3)	0.060 (2)	0.059 (2)	−0.015 (2)	−0.035 (2)	−0.0203 (18)
C9	0.064 (2)	0.062 (2)	0.0488 (18)	−0.0043 (17)	−0.0251 (17)	−0.0193 (16)
C10	0.101 (4)	0.113 (4)	0.052 (2)	−0.022 (3)	−0.027 (2)	−0.028 (2)
C11	0.0494 (17)	0.0335 (14)	0.0522 (17)	−0.0071 (13)	−0.0011 (14)	−0.0168 (13)
C12	0.0470 (17)	0.0408 (16)	0.0516 (17)	−0.0059 (13)	−0.0046 (13)	−0.0230 (13)
C13	0.0398 (15)	0.0447 (16)	0.0443 (15)	−0.0046 (12)	−0.0067 (12)	−0.0174 (13)
C14	0.061 (2)	0.0338 (15)	0.0496 (17)	−0.0106 (14)	−0.0003 (15)	−0.0110 (13)
C15	0.062 (2)	0.0337 (15)	0.0469 (16)	−0.0083 (14)	0.0011 (14)	−0.0185 (13)
C16	0.0557 (19)	0.0467 (17)	0.0467 (17)	−0.0092 (15)	−0.0036 (14)	−0.0162 (14)

### Geometric parameters (Å, °)

**Table:**

U1—O1	1.772 (2)	C5—H5A	0.9600
U1—O2	1.768 (2)	C5—H5B	0.9600
U1—O3	2.374 (2)	C5—H5C	0.9600
U1—O4	2.314 (2)	C6—C7	1.509 (5)
U1—O5	2.342 (2)	C6—H6A	0.9600
U1—O6	2.350 (2)	C6—H6B	0.9600
U1—N1	2.599 (3)	C6—H6C	0.9600
O3—C4	1.275 (4)	C7—C8	1.383 (6)
O4—C2	1.272 (4)	C8—C9	1.382 (6)
O5—C9	1.272 (4)	C8—H8	0.9300
O6—C7	1.260 (4)	C9—C10	1.500 (6)
O7—N2	1.394 (4)	C10—H10A	0.9600
O7—H7	0.8200	C10—H10B	0.9600
N1—C15	1.334 (4)	C10—H10C	0.9600
N1—C11	1.345 (4)	C11—C12	1.367 (4)
N2—C16	1.259 (5)	C11—H11	0.9300
C1—C2	1.499 (5)	C12—C13	1.394 (4)
C1—H1A	0.9600	C12—H12	0.9300
C1—H1B	0.9600	C13—C14	1.372 (4)
C1—H1C	0.9600	C13—C16	1.462 (4)
C2—C3	1.377 (5)	C14—C15	1.382 (5)
C3—C4	1.389 (5)	C14—H14	0.9300
C3—H3	0.9300	C15—H15	0.9300
C4—C5	1.497 (5)	C16—H16	0.9300
			
O1—U1—O2	177.73 (10)	C4—C5—H5A	109.5
O1—U1—O3	94.67 (10)	C4—C5—H5B	109.5
O1—U1—O4	89.96 (10)	H5A—C5—H5B	109.5
O1—U1—O5	90.77 (11)	C4—C5—H5C	109.5
O1—U1—O6	93.99 (11)	H5A—C5—H5C	109.5
O1—U1—N1	86.40 (10)	H5B—C5—H5C	109.5
O2—U1—O3	86.31 (10)	C7—C6—H6A	109.5
O2—U1—O4	88.42 (10)	C7—C6—H6B	109.5
O2—U1—O5	91.46 (11)	H6A—C6—H6B	109.5
O2—U1—O6	86.34 (11)	C7—C6—H6C	109.5
O2—U1—N1	91.57 (10)	H6A—C6—H6C	109.5
O3—U1—O4	71.32 (8)	H6B—C6—H6C	109.5
O3—U1—O5	75.30 (8)	O6—C7—C8	123.4 (3)
O3—U1—O6	145.01 (9)	O6—C7—C6	115.5 (4)
O3—U1—N1	142.48 (8)	C8—C7—C6	121.1 (3)
O4—U1—O5	146.56 (8)	C9—C8—C7	125.1 (3)
O4—U1—O6	142.51 (8)	C9—C8—H8	117.4
O4—U1—N1	71.18 (8)	C7—C8—H8	117.4
O5—U1—O6	70.74 (8)	O5—C9—C8	123.3 (3)
O5—U1—N1	142.22 (8)	O5—C9—C10	116.0 (4)
O6—U1—N1	71.89 (8)	C8—C9—C10	120.6 (3)
C4—O3—U1	133.1 (2)	C9—C10—H10A	109.5
C2—O4—U1	131.5 (2)	C9—C10—H10B	109.5
C9—O5—U1	138.1 (2)	H10A—C10—H10B	109.5
C7—O6—U1	137.8 (2)	C9—C10—H10C	109.5
N2—O7—H7	109.5	H10A—C10—H10C	109.5
C15—N1—C11	117.3 (3)	H10B—C10—H10C	109.5
C15—N1—U1	121.6 (2)	N1—C11—C12	123.6 (3)
C11—N1—U1	121.0 (2)	N1—C11—H11	118.2
C16—N2—O7	111.2 (3)	C12—C11—H11	118.2
C2—C1—H1A	109.5	C11—C12—C13	118.7 (3)
C2—C1—H1B	109.5	C11—C12—H12	120.6
H1A—C1—H1B	109.5	C13—C12—H12	120.6
C2—C1—H1C	109.5	C14—C13—C12	118.0 (3)
H1A—C1—H1C	109.5	C14—C13—C16	119.6 (3)
H1B—C1—H1C	109.5	C12—C13—C16	122.4 (3)
O4—C2—C3	123.3 (3)	C13—C14—C15	119.8 (3)
O4—C2—C1	115.3 (3)	C13—C14—H14	120.1
C3—C2—C1	121.3 (3)	C15—C14—H14	120.1
C2—C3—C4	125.1 (3)	N1—C15—C14	122.6 (3)
C2—C3—H3	117.5	N1—C15—H15	118.7
C4—C3—H3	117.5	C14—C15—H15	118.7
O3—C4—C3	123.4 (3)	N2—C16—C13	120.8 (3)
O3—C4—C5	116.4 (3)	N2—C16—H16	119.6
C3—C4—C5	120.2 (3)	C13—C16—H16	119.6

### Hydrogen-bond geometry (Å, °)

**Table:**

*D*—H···*A*	*D*—H	H···*A*	*D*···*A*	*D*—H···*A*
O7—H7···O5^i^	0.82	2.49	3.018 (4)	123
O7—H7···O3^i^	0.82	2.29	3.083 (4)	163

Symmetry codes: (i) *x*−1, *y*, *z*+1.
